# Universal scaling of weak localization in graphene due to bias-induced *dispersion decoherence*

**DOI:** 10.1038/s41598-020-62313-3

**Published:** 2020-03-27

**Authors:** R. Somphonsane, H. Ramamoorthy, G. He, J. Nathawat, S. Yin, C.-P. Kwan, N. Arabchigavkani, B. Barut, M. Zhao, Z. Jin, J. Fransson, J. P. Bird

**Affiliations:** 1 Department of Physics, King Mongkut’s Institute of Technology Ladkrabang, Bangkok, 10520 Thailand; 2 Thailand Center of Excellence in Physics, Commission on Higher Education, 328 Si Ayutthaya Road, Bangkok, 10400 Thailand; 3 Department of Electronic Engineering, King Mongkut’s Institute of Technology Ladkrabang, Bangkok, 10520 Thailand; 4 Department of Electrical Engineering, University at Buffalo, The State University of New York, Buffalo, NY 14260-1900 USA; 5 Department of Physics, University at Buffalo, The State University of New York, Buffalo, NY 14260-1500 USA; 6 High-Frequency High-Voltage Device and Integrated Circuits Center, Institute of Microelectronics of Chinese Academy of Sciences, 3 Beitucheng West Road, Chaoyang District, Beijing, PR China; 7 Department of Physics and Astronomy, Uppsala University, Box 516, SE-751 21 Uppsala, Sweden

**Keywords:** Condensed-matter physics, Electronic properties and materials

## Abstract

The differential conductance of graphene is shown to exhibit a zero-bias anomaly at low temperatures, arising from a suppression of the quantum corrections due to weak localization and electron interactions. A simple rescaling of these data, free of any adjustable parameters, shows that this anomaly exhibits a universal, temperature- (*T*) independent form. According to this, the differential conductance is approximately constant at small voltages (*V* < *k*_*B*_*T*/*e*), while at larger voltages it increases logarithmically with the applied bias. For theoretical insight into the origins of this behaviour, which is inconsistent with electron heating, we formulate a model for weak-localization in the presence of nonequilibrium transport. According to this model, the applied voltage causes unavoidable *dispersion decoherence*, which arises as diffusing electron partial waves, with a spread of energies defined by the value of the applied voltage, gradually decohere with one another as they diffuse through the system. The decoherence yields a universal scaling of the conductance as a function of *eV*/*k*_*B*_*T*, with a logarithmic variation for *eV*/*k*_*B*_*T* > 1, variations in accordance with the results of experiment. Our theoretical description of nonequilibrium transport in the presence of this source of decoherence exhibits strong similarities with the results of experiment, including the aforementioned rescaling of the conductance and its logarithmic variation as a function of the applied voltage.

## Introduction

It has long been understood that the conductance of mesoscopic systems may exhibit quantum corrections at low temperatures, arising from the combined influence of weak localization^1–3^ and electron interactions^3–5^. The former phenomenon^1^ is due to the coherent interference of time-reversed pairs of Feynman paths, which return to their origin after a sequence of elastic scattering events, thereby enhancing the resistance above its Drude value. The interaction correction^2^, on the other hand, has been discussed in terms of the scattering generated by the charge hologram associated with such closed paths^5^. While these corrections have long been the subject of study in normal metals and semiconductors, interest in these phenomena has been revived in recent years due to their manifestations in emergent two-dimensional materials, with the most notable example being provided by graphene. The unusual aspects of the bandstructure of this material, including its linear energy dispersion and the chiral nature of its carriers, significantly modify weak localization, in a manner that has been discussed in a number of theoretical^6–12^ and experimental^13–24^ works. Most notable here is that the details of the localization are strongly dependent upon the nature of the impurities in the system, with exact backscattering being forbidden for remote impurities that generate long-range scattering^25,26^. As such, this behaviour corresponds to weak antilocalization, a phenomenon that is normally associated with materials with strong spin-orbit coupling^1–3^. The antilocalization occurs in spite of the very weak spin-orbit coupling in graphene, but is suppressed in the presence of short-ranged impurities; these restore weak localization by allowing backscattering between the inequivalent *K* and ${K}^{{\prime} }$ valleys. Elsewhere, other works have explored the nature of the interaction-related correction to graphene’s conductance^27–32^, and have found it to be of similar magnitude to that arising from weak localization.

In both the original experimental work on quantum corrections in normal metals and semiconductors^1–3,5^, and in more recent investigations performed on graphene^13–24,27–32^, the primary emphasis has been on obtaining information on these phenomena by studying their influence on the equilibrium charge transport conductance. More specifically, most works have addressed the manner in which the corrections are affected by a magnetic field, which breaks time-reversal symmetry and suppresses weak localization while leaving the interaction contribution unaffected^1–5^. In contrast, far fewer studies have explored the manner in which these phenomena are affected under nonequilibrium conditions. (Notable exceptions include early works that demonstrated the inability of an electric field to break time reversal during coherent backscattering^2,33,34^, and later experiments on the differential conductance of GaAs/AlGaAs quantum dots^35^ and short metallic nanobridges^36,37^.) While there have been a few investigations of the nonlinear differential conductance of graphene^38–43^, there is still relatively little that is understood about the manner in which the quantum corrections in this material (and in other Dirac materials) are affected under nonequilibrium conditions. It is this specific problem that we address here, from both experimental and theoretical perspectives.

The experimental component of this work involves studies of the differential conductance (*g*) of graphene transistors, implemented in both monolayer and bilayer material. At low temperatures (*T*), where quantum corrections are expected to influence transport, the zero-bias conductance (*G*) of these devices is suppressed by the combined influence of weak localization and electron interactions. Application of a nonzero voltage (*V*) quenches these phenomena, however, and leads to an enhancement of the differential conductance that defines a zero-bias anomaly. By implementing a simple rescaling of these data, in which we plot the bias-induced change of differential conductance (*Δg* = *g*(*V*) − *G*) as a function of the dimensionless voltage (*eV*/*k*_*B*_*T*, where *k*_*B*_ is the Boltzmann constant), we show that the zero-bias anomaly collapses onto a universal, temperature-independent form. According to this, the linear conductance remains unchanged for voltages *eV* ≲ *k*_*B*_*T*, while at larger voltages it increases as a logarithmic function of *V*, reflecting the quenching of the quantum corrections. This universal voltage scaling of the quantum corrections is observed in both monolayer and bilayer devices, and on the electron and hole sides of the Dirac point. Quantitative insight into the origins of this behaviour, which cannot be explained in terms of current-induced heating, is provided in the theoretical component of this work, in which we develop a formal description of the weak-localization correction under strongly nonequilibrium conditions. This is achieved by making use of a nonequilibrium Green function approach, in which we address the influence of disorder-induced scattering up to the level of the maximally-crossed diagrams responsible for weak localization. Our essential finding is that the applied voltage introduces an additional dephasing in transport, which suppresses localization in a manner analogous to other sources of decoherence^44^. More specifically, while it does not break time reversal symmetry, by opening an energy window for transport, the applied voltage causes a *dispersion decoherence*. According to this, diffusing electron waves, with a spread of energies set by the value of the voltage, gradually decohere with one another as they propagate around the same scattering loop. While our calculations are performed for the weak-localization correction alone, the strong similarities that they exhibit with the results of our experiment suggest that the interaction correction should be similarly affected by the self-averaging phenomenon.

## Results

### Experimental methods

**Figure 1:**
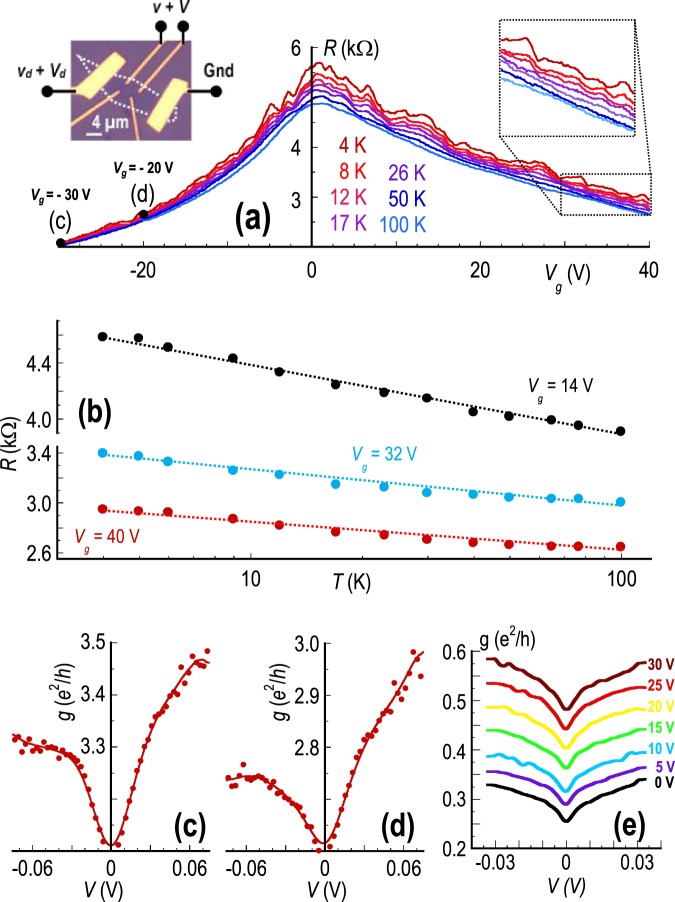
(**a**) The main panel shows the variation of the small-signal (AC) resistance as a function of gate voltage, for the bilayer device B1 shown in the optical image in the left inset. This inset also indicates the biasing scheme used to measure differential conductance in a four-probe configuration. The right inset is an expanded view of the data, indicating the emergence of conductance fluctuations and the quantum corrections at low temperatures. (**b**) Variation of resistance as a function of temperature, determined from the results of panel (**a**) at three different gate voltages (indicated). The dotted lines are guides to the eye that indicate the logarithmic scaling characteristic of quantum corrections. Note the break in the vertical axis of the figure. (**c**,**d**) Measurements of the differential conductance of device B1 at 4 K and for the two gate voltages identified in the main panel (**a**). Filled symbols are experimental data while the solid lines represent weighted fits through these points. (**e**) Similar differential conductance data obtained from device B2 at several gate voltages spanning the electron densities. The Dirac point for this device is at  −5 V.

Graphene devices were fabricated by exfoliating Kish graphite onto a heavily-doped Si substrate with a 300-nm SiO_2_ cap layer^45,46^. Layer identification was achieved through a combination of optical microscopy and Raman imaging^46^, following which individual graphene flakes were contacted with Cr/Au (3-/50-nm) electrodes, defined by electron-beam lithography and lift-off. The conductive Si substrate served as the (back-) gate of these devices, which was biased at an appropriate voltage (*V*_*g*_) to vary the carrier concentrations. A number of devices were fabricated, and characterized electrically, and exhibited similar and consistent characteristics. In this work we focus on a detailed study of the differential conductance of representative devices (see Table 1) realized from monolayer and bilayer graphene. The measured electron (*μ*_*e*_) and hole (*μ*_*h*_) mobilities in these devices at 4 K and a concentration of 10^12^ cm^−2^ were consistent with prior reports^47^ for graphene on SiO_2_. For four-probe measurements of the linear conductance (*G*), a small AC voltage (*v*_*d*_) was applied between the source and drain contacts of the device, allowing the conductance to be determined from the measured AC current (*i*_*d*_) and from the voltage (*v*) drop across a pair of internal voltage probes. An example of this configuration is provided in the left inset of Fig. 1(a), which features an optical micrograph of one of our bilayer devices. The value of *v*_*d*_ was configured to yield an internal voltage *v* ~ 100*μ*V and the measurement frequency was set at 13 Hz. For measurements of the differential conductance, the AC conductance was determined in the same manner as above, but now with an additional DC voltage (*V*_*d*_, applied to the source and drain contacts) superimposed upon the AC component. In all of the data presented here, the variation of differential conductance is indicated as a function of the DC voltage drop (*V*) across the pair of internal probes. This voltage was determined from the measured AC voltages, according to (*V* = *V*_*d*_ ⋅ (*v*/*v*_*d*_)). All measurements were made with the samples mounted in vacuum, on the cold finger of a closed-cycle cryostat.

**Table 1:** Important parameters for the different devices studied.

Device	*L*^*a*^(*μm*)	*W*^*a*^(*μm*)	${\mu }_{h}^{b}(c{m}^{2}/Vs)$	${\mu }_{e}^{b}(c{m}^{2}/Vs)$	${\sigma }_{min}^{c}({e}^{2}/h)$	${l}_{h}^{d}(nm)$	${l}_{e}^{d}(nm)$
M1	1.1	1.3	14200	11600	22	166	135
B1	1.5	1	2300	1800	5.5	27	21
B2	4	0.25	1100	1280	4	13	15

^*a*^*L*, *W*:  channel length, width, measured between voltage probes. ^*b*^*μ*_*e*_, *μ*_*h*_:  Electron, hole mobility, determined at 4 K, and at a density of 10^12^*cm*^−2^.^*c*^*σ*_*min*_:  minimum conductivity, measured at 4 K. ^*d*^*l*_*e*_, *l*_*h*_:  Electron, hole mean free path, determined at 4 K, and at density of 10^12^*cm*^−2^.

### Experimental results

We begin our discussions by focusing on the manner in which the quantum corrections are manifested in the linear-transport characteristics of the devices. In the main panel of Fig. 1(a), we show the variation of the small-signal resistance (*R* ≡ 1/*G*) of device B1 as a function of its gate voltage. Measurements are shown for various temperatures from 4  −  100 K, and reveal several important characteristics. Firstly, as the temperature is lowered below 30 K, reproducible conductance fluctuations develop in the curves, signaling the emergence of coherent mesoscopic transport^48,49^. More importantly, the fluctuations are superimposed upon a background trend for increasing resistance with decreasing temperature (see the right inset to Fig. 1(a)), behaviour that is typical of the quantum corrections^1–5^. This connection is further established in Fig. 1(b), where we plot the variation of resistance as a function of temperature at three representative gate voltages. In all three cases, the resistance varies as a logarithmic function of temperature, a characteristic signature of quantum corrections in two-dimensional materials^1–5^.

The main emphasis in this report is on understanding the manner in which the quantum corrections in graphene are suppressed under nonequilibrium conditions. The essential phenomenon in which we will be interested is presented in Figs. 1(c,d), in which we plot the low-temperature (4 K) differential conductance of device B1 at two gate voltages (identified in the main panel of Fig. 1(a)) and Fig. 1(e), in which we plot similar curves obtained from device B2 spanning several gate voltages. Common to these data is a zero-bias anomaly, according to which the conductance is suppressed at zero bias but then increases when a voltage of either polarity is applied. (The anomaly is superimposed upon a broader conductance variation, which has previously been discussed as a signature of electron heating^42^.) As we now describe, the zero-bias anomaly is attributed to a nonequilibrium suppression of the quantum corrections in these devices.

**Figure 2:**
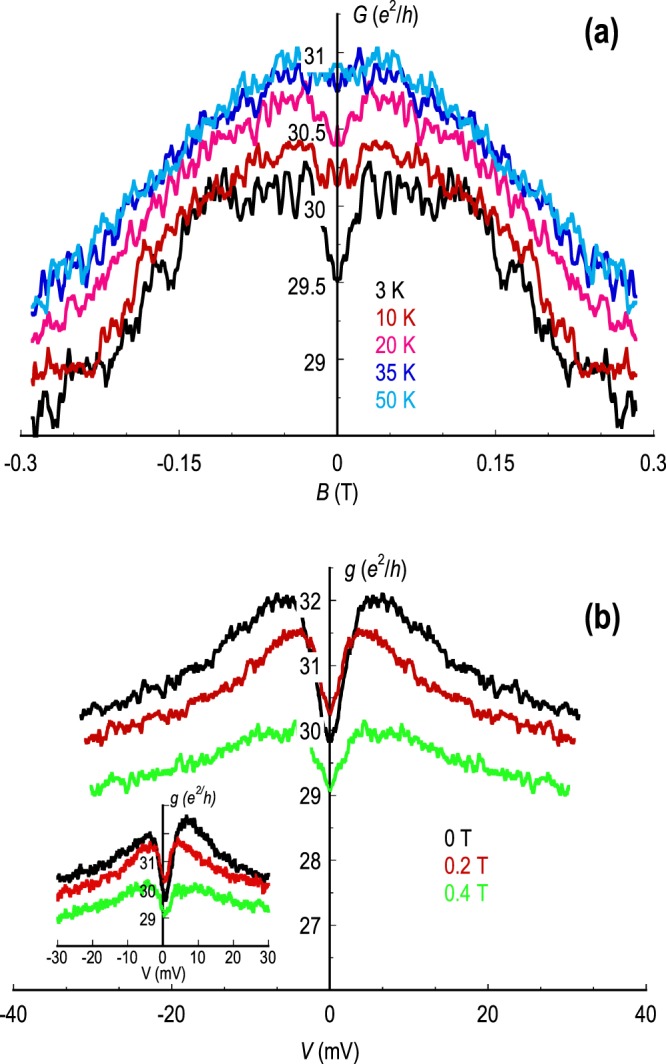
(**a**) Symmetric component^46^ of the linear magneto-conductance of the monolayer device M1 at five different temperatures (indicated). (**b**) Differential conductance of the monolayer device at 3 K and at three magnetic fields. The inset shows the corresponding raw, un-symmetrized data. Data in panels (**a**) and (**b**) were obtained for a gate voltage at the Dirac point of the device (*V*_*g*_ = 14 V).

In normal investigations of quantum corrections, it is common to distinguish the influence of weak localization and electron interactions by applying a perpendicular magnetic field (*B*); as noted already, this suppresses weak localization while leaving the interaction correction unaffected^1–5^. In Fig. 2(a), we show the results of measurements of the symmetric component^46^ of the linear magneto-conductance (*G*_*sym*_(±*B*) = (*G*( + *B*) + *G*( − *B*))/2) of the monolayer device M1 at five different temperatures. At 3 K, the signature of weak localization can be clearly seen in the data, in the form of a narrow conductance dip that is centered at zero magnetic field, and which coexists with reproducible fluctuations that extend over the entire field range. Both of these features weaken as the temperature is increased to 35 K, consistent with the expected reduction in carrier phase coherence^44,48,49^. The most important point here, however, is that the localization is quenched at very weak magnetic fields, as little as 30 mT. Noting this, in Fig. 2(b) we plot the results of measurements of the low-temperature differential conductance of the monolayer device at three different magnetic fields. These data were obtained for a gate voltage close to the Dirac point and at *B* = 0 T the zero-bias anomaly is close to 2*e*^2^/*h* in size. Its amplitude is reduced under the application of the magnetic field, decreasing to around 50 % of its original value at 0.4 T. The crucial point here is that this field value is significantly larger than that required in Fig. 2(a) to suppress weak localization. Consequently, we may conclude that the zero-bias anomaly in the differential conductance arises, at zero magnetic field at least, from the combined influence of weak localization and electron interactions. Moreover, the data of Fig. 2(b) suggests that the relative magnitude of these two quantum corrections should be roughly the same. While the data of Fig. 2(a,b), were obtained for a monolayer device, with the gate voltage configured at the Dirac point, the general features of the zero-bias anomaly revealed in these figures were found also in our studies of bilayer graphene (as confirmed already in Fig. 1), and were largely unchanged as the gate voltage was used to move the Fermi level between the valence and conduction bands.

**Figure 3:**
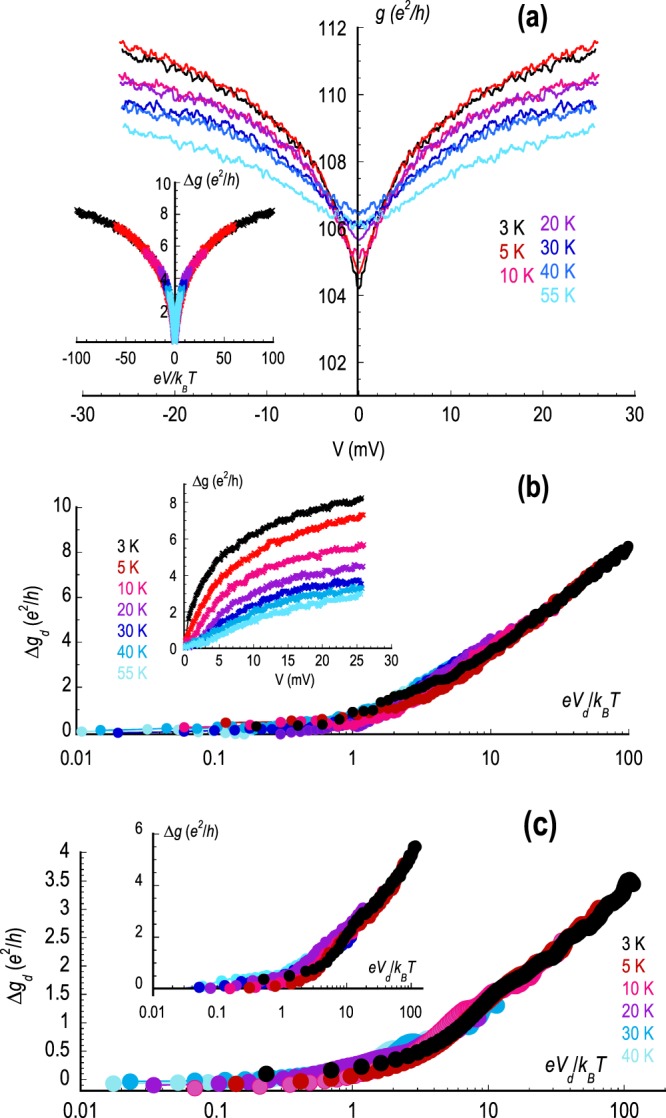
(**a**) The main panel shows the results of measurements of the differential conductance of device M1 at various temperatures from 3–55 K. The data were obtained for a gate voltage *V*_*g*_ = −6 V, while the Dirac point in this device was positioned at *V*_*g*_ = + 14 V. This condition therefore corresponds to a gate-induced hole concentration of *p* = 1.44 ⋅ 10^12^ cm^−2^. The inset shows a rescaling of the data from the main panel, according to which we plot the variation of the bias-induced conductance change (*Δg* = *g*(*V*) − *G*) as a function of the dimensionless voltage (*eV*/*k*_*B*_*T*). The colors of the different data points correspond to the same temperatures as indicated in the main panel. (**b**) Data from the inset of (**a**) replotted with the dimensionless voltage represented on a logarithmic scale to highlight the collapse of the various curves on top of each other. The inset shows the conductance change as a function of the unscaled bias voltage. (**c**) Similar results as (**b**) obtained at *V*_*g*_ = + 22 V, corresponding to an electron concentration of *n* = 5.76 ⋅ 10^11^ cm^−2^ and at *V*_*g*_ = +6 V (inset) corresponding to a hole concentration of *p* = 5.76 ⋅ 10^11^ cm^−2^.

Consistent with the temperature-dependent nature of linear transport, as evidenced in Figs. 1, 2, the zero-bias anomaly in the differential conductance is also found to depend strongly upon temperature. This is illustrated in Fig. 3(a), in the main panel of which we show measurements of the differential conductance of the monolayer device M1 at a number of different temperatures and at a fixed gate voltage of *V*_*g*_ = − 6 V, which corresponds to a gate-induced hole concentration of *p* = 1.44 ⋅ 10^12^ cm^−2^. As the temperature is increased from an initial value of 3 K, two important trends are apparent in this data: firstly, the zero-bias conductance increases with increasing temperature, consistent with the expected suppression of the quantum corrections, and; secondly, the overall amplitude of the zero-bias anomaly is simultaneously reduced. Remarkably, we find that these data can be rescaled onto a common curve by implementing the procedure utilized in the inset to Fig. 3(a). Here we replot the data of the main panel to show the variation of the bias-induced conductance change (*Δg* = *g*(*V*) − *G*) as a function of the dimensionless voltage (*eV*/*k*_*B*_*T*). Simply by means of this parameter-free rescaling, we see that the conductance curves obtained at different temperatures essentially collapse onto one another. This is further highlighted in the main panel of Fig. 3(b) where the data is replotted with the dimensionless voltage indicated on a logarithmic scale. From these data, it is clear that the differential conductance exhibits two distinct regimes of voltage-dependent behaviour; the first at low voltages where it is approximately constant, and the second at higher voltages where it instead exhibits a logarithmic variation. The universality of the observed logarithmic scaling behaviour is further demonstrated in Fig. 3(c) (and its inset) where we see similar collapse of the conductance curves at other carrier concentrations spanning the electron and hole spectrum of the monolayer device M1.

**Figure 4:**
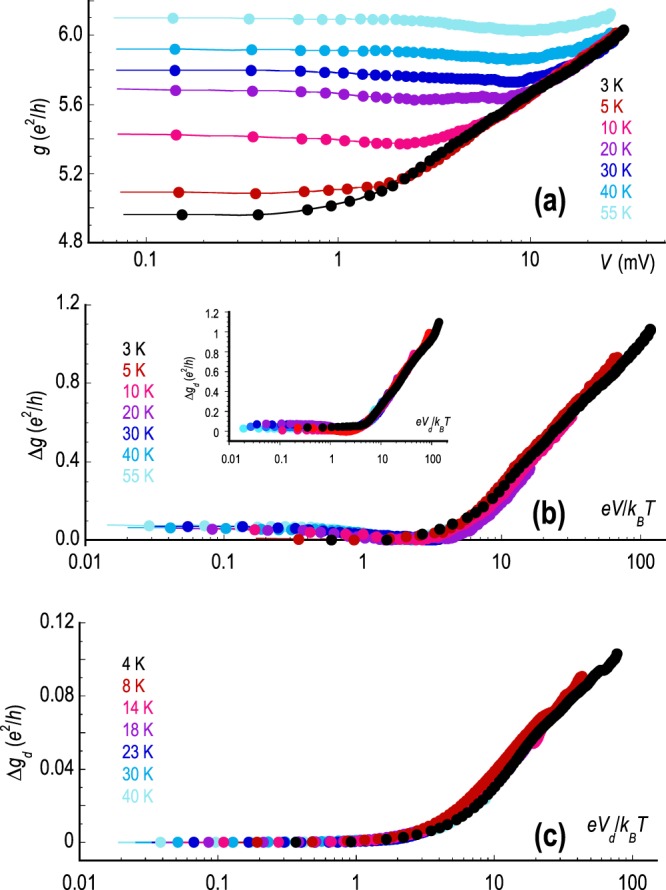
Temperature dependent differential conductance of device B1, measured at various temperatures from 3–55 K. The data were obtained for a gate voltage *V*_*g*_ = −24 V, while the Dirac point in this device was positioned at *V*_*g*_ = + 2 V. This condition therefore corresponds to a gate-induced hole concentration of *p* = 1.87 ⋅ 10^12^ cm^−2^. (**b**) Data of panel (**a**), obtained after rescaling. The inset shows the rescaled data at *V*_*g*_ = −6 V which corresponds to *p* = 5.76 ⋅ 10^11^ cm^−2^. (**c**) Similar rescaling obtained for device B2 at a gate voltage *V*_*g*_ = −40 V (hole concentration *p* = 2.52 ⋅ 10^12^ cm^−2^, Dirac point in this device is at *V*_*g*_ = −5 V).

For further insight into the rescaling of the differential conductance, we refer to the results of Fig. 4. In contrast to the results of Fig. 3, which were obtained for the monolayer device M1, we now show the results of this rescaling for the bilayer devices investigated here. In Fig. 4(a), we show the variation of the differential conductance (*g*(*V*)) for device B1 as a function of the applied voltage (with the abscissa indicated on a logarithmic scale) and at a gate voltage *V*_*g*_ = −24 V, which corresponds to a hole concentration of *p* = 1.87 ⋅ 10^12^ cm^−2^. From these data alone, it is evident that the two-distinct regimes of voltage-dependent behaviour, observed in Fig. 3, is also present in the bilayer device shown here. Fig. 4(b), shows the data of Fig. 4(a), but with the abscissa now indicating the dimensionless voltage instead. As with the behaviour revealed in Fig. 3(b,c) we once again see how the curves obtained at various temperatures in Fig. 4(a) collapse onto a common curve as a result of the data rescaling. This behaviour is further evident from the data shown in the inset to Fig. 4(b) where similar results are obtained at a different carrier concentration. Having replotted the data in this way, it is moreover apparent that the crossover between the voltage-independent and voltage-dependent regimes occurs for *eV*/*k*_*B*_*T* ~ 1, a natural result if the influence of the voltage is to open a thermally-resolved energy window for transport (a point that we return to below). In Fig. 4(c), we provide yet another illustration of this rescaling, in this case for the device B2. The similarity of these results to those of Figs. 4(b) and 3(b,c) is striking, with a crossover near *eV*/*k*_*B*_*T* ~ 1 and a logarithmic scaling at higher voltages.

Having established the universal voltage scaling of the quantum corrections in graphene, in the next section we develop a theoretical model to account for this behaviour. Our approach involves formulating a description of the weak-localization correction under nonequilibrium, and demonstrating that this exhibits the essential characteristics of our experimental data. While a full treatment of this problem should also involve calculating the interaction-induced correction under nonequilibrium, this latter task is considered to be beyond the scope of the current work. By clarifying how weak localization is influenced under nonequilibrium, however, we gain important insight into the relevant processes responsible for the quenching of the quantum corrections in experiment.

### Theoretical treatment of weak localization in graphene under nonequilibrium

The main objective in this section is to present a theory for weak localization in graphene that is valid under nonequilibrium conditions, and which is able to capture the quantitative features of our experiment. As a first step towards the development of this theory, we begin by making some general statements about the need to go beyond standard (Cooperon-based) descriptions, which have been shown to lead to the result that the localization correction is unaffected by a static electric field. By instead implementing a fully-nonequilibrium approach, we show that this result is not generally valid, and that the localization correction may in fact be affected by such a field. This motivates us to approach the development of our theory from a more general perspective that allows us to fully account for the presence of the electric field.

### The influence of an electric field on weak localization

In usual discussions of weak localization it is common to describe this phenomenon in terms of the two-particle propagator known as the Cooperon^2,3,50,51^. This comprises a quasiparticle that follows a diffusive path, and which interferes with its time-reversed partner, providing the natural viewpoint from which to discuss weak localization. As a linear-response construct, however, the Cooperon may only be utilized under equilibrium, or quasiequilibrium, conditions. The extension of this problem to nonequilibrium was considered in a number of early theoretical works^52–56^ (see Bergmann^57,58^ for further discussion), with very different predictions. Even in the absence of any field-induced heating, Tsuzuki^52–54^ suggested that the localization should be suppressed by a static electric field, a result that was contradicted by Altshuler and Aronov^55,56^. They argued that the Cooperon is unaffected by a static field, and that the localization correction should therefore be similarly unchanged. This result has been discussed by Bergmann^57^, who notes that it basically follows from the fact that Cooperon is determined by summing over a number of terms, each of which involves the product of a retarded and an advanced Green function. Even under nonequilibrium, the field-dependent terms in the these propagators are found to cancel one another exactly, indicating that there is no influence of the field on the localization^57^. In subsequent experiments performed on thin metal films, Bergmann showed that the quantum corrections were indeed invariant to the application of electric fields as large as 10^2^ V/m, appearing to confirm the predictions of Altshuler and Aronov.

In spite of the discussion above, it has previously been noted that an inconsistency in the arguments of refs. ^55,56^ arises from the fact that they are based on an appeal to the Kubo formula. As Bergmann himself has pointed out^57^, it is questionable whether conclusions pertaining to nonequilibrium transport may be accurately drawn from such a linear-response theory. Specifically, *it is well known that the Kubo formalism cannot be utilized to consistently treat the conductance in the presence of an external electric field*. Consequently, it is necessary to adopt an alternative approach to this problem if nonequilibrium effects are to be fully accounted for.

In the Supplementary Information (SI) of this paper, we present a quite general approach to computing the weak-localization correction under the influence of a static electric field, in which we start from an expression for the current operator^59^:

1 $${\bf{j}}({\bf{r}},t)\sim (-i)\mathop{lim}\limits_{{{\bf{r}}}^{{\rm{{\prime} }}}\to {\bf{r}}}({{\rm{\nabla }}}_{{\bf{r}}}-{{\rm{\nabla }}}_{{{\bf{r}}}^{{\rm{{\prime} }}}}){{\bf{G}}}^{ < }({\bf{r}},{{\bf{r}}}^{{\rm{{\prime} }}};t,{t}^{{\rm{{\prime} }}}).$$

Here, ${{\bf{G}}}^{ < }({\bf{r}},{{\bf{r}}}^{{\rm{{\prime} }}};t,{t}^{{\rm{{\prime} }}})$ is the lesser form of the single-electron Green function, describing the propagation of a particle from an initial position **r** (at time *t*) to position ${{\bf{r}}}^{{\prime} }$ at time ${t}^{{\prime} }$. Working within this framework, we show that calculation of the weak-localization correction now requires the treatment of terms involving the product of (a minimum of) three Green functions, rather than the two-function products involved in the Cooperon description. These terms are of the form ${{\bf{G}}}_{0}^{r}{{\bf{G}}}_{0}^{ < }{{\bf{G}}}_{0}^{a}$, involving the retarded, lesser, and advanced forms, respectively, of the bare Green function (**G**_0_) of the undisordered system. In contrast to the Cooperon-based treatment, the field-dependent terms do *not* cancel in these products, but rather yield complicated integrals in which the electric field remains (see the SI). While these integrals preserve time-reversal symmetry, they may nonetheless give rise to a nontrivial influence of the electric field on localization.

### A nonequilibrium model for weak localization in graphene

Having recognized the issues above, in this section we develop an alternative approach to the treatment of weak localization^60–62^ that is based on the use of nonequilibrium Green functions. Under spatial averaging, we obtain *rainbow* diagrams that provide the impurity-limited scattering lifetime in the self-consistent Born approximation, and *maximally-crossed* diagrams that account for weak localization (see the SI). To implement our calculations, we consider a graphene sheet contacted by a pair of metallic leads, and calculate its electronic structure subject to the boundary conditions imposed by the coupling to these leads. To consider the details of transport in the presence of disorder, we introduce short-range scattering centers that represent atomically-sharp defects. By including such impurities in our model we are able to capture effects arising from inter-valley scattering^63,64^, a key mechanism that is necessary to give rise to weak localization^6^. (The role of long-range disorder, such as that generated by substrate impurities, is not considered here. In this sense, our analysis is pertinent to intrinsic graphene, in the presence of local imperfections and defects, but free of any substrate interactions.)

While the details of our calculation of the nonequilibrium weak-localization correction are provided in the SI to this paper, the essential features of our theory are as follows. Using a tight-binding model of the monolayer graphene lattice, with imperfections arising from a random collection of short-ranged impurities, and tunneling to a pair of metallic leads, we calculate the current flowing through this layer under the application of fixed voltage. With the nonequilibrium nature of this problem taken into account by formulating the model on the Keldysh contour (see the SI), the reduction of current (*δI*) due to weak-localization is determined by applying an impurity averaging, in which the contribution to the current from the maximally-crossed diagrams is calculated. Using the resulting expression, the localization-induced correction to the differential conductance is ultimately expressed (in the limit *T* → 0) as:

2 $$\frac{d}{dV}\delta I=-\,\frac{2{e}^{2}}{h}\frac{{\Gamma }^{L}{\Gamma }^{R}}{{D}_{c}^{2}}{\rm{ln}}\,\frac{{D}_{c}}{eV}.$$

Here, *Γ*^*L*,*R*^ represent the coupling between the graphene and its left and right reservoirs (we take *Γ*^*L*^ = *Γ*^*R*^ = 5 meV), ${D}_{c}={(4\pi {v}_{F}2\rho )}^{1/2}=3$ eV is an upper energy limit, *v*_*F*_ is the Fermi velocity of graphene, and *ρ* is its planar density^65,66^. Crucially, Eq. ((2)) indicates that the localization correction to the differential conductance decreases with increasing voltage, exhibiting a logarithmic scaling as a function of this parameter that is highly suggestive of that found in our experiment.

**Figure 5:**
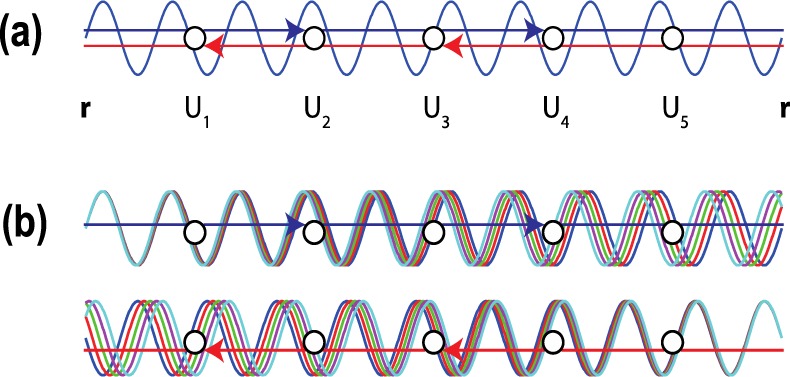
A schematic illustration of the energy averaging of weak localization due to a nonzero voltage. (**a**) At zero voltage, weak localization arises from the interference of monoenergetic partial waves that travel in opposite directions. An electron starts at point r and returns to the same point after a sequence of (five) scattering events. *U*_1−5_ denote the different potentials associated with these scatterers. (**b**) At non-zero voltage, the monoenergetic waves in (**a**) are replaced by a set of waves with a spread of energies, which decohere with one another over an effective, voltage-induced, decoherence length. In this mechanism of dispersion decoherence, the essential point is that these waves are initially coherent as they commence their diffusion, but that they end up with a variety of phases by the time that they return to the origin. This results in destructive interference and a suppression of the localization correction.

Before undertaking a quantitative comparison of our experiment and theory, we comment on the significance of Eq. (2). Physically, this describes an additional source of dephasing, a dispersion decoherence, whose essential idea is as follows. At zero temperature, and in the absence of any magnetic field or applied voltage, there is a fixed phase difference between electron waves that traverse any scattering loop in opposite directions. Increasing temperature leads to a summation over several such loops, each with the same fixed phase difference, so that the constructive interference that is the origin of weak localization is maintained. (This is the well-known statement that weak localization is not subject to thermal averaging^3^.) When a nonzero voltage is now applied, however, each diffusing electron essentially corresponds to a set of partial waves, with a spread of energies determined by the value of the applied voltage. As these waves diffuse through the graphene sheet, the dispersion due to their energy spread leads to a natural dephasing, as indicated in Fig. 5.

**Figure 6:**
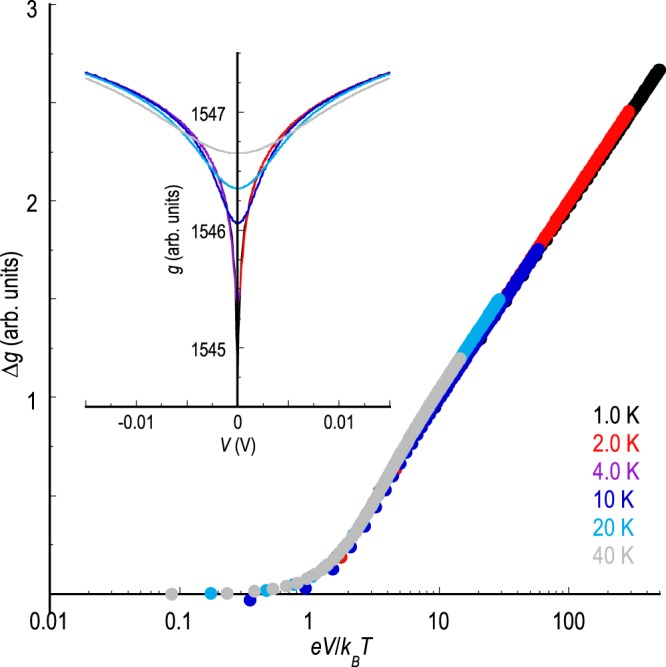
In the inset to this figure, we show the calculated differential conductance for monolayer graphene (temperatures indicated) in the presence of weak localization. The impurity concentration is set at 1 % and the scattering potential associated with the impurities is 1 eV. In the main panel we replot the differential conductance data to show the variation of bias induced conductance change (*Δg*) as a function of the dimensionless voltage (*eV*/*k*_*B*_*T*).

In the inset to Fig. 6, we plot the calculated contribution of weak localization to the differential conductance of graphene, over a temperature range similar to that studied in experiment. In these calculations, the impurity concentration is taken to be 1 % and the scattering potential associated with the impurities (*U*_*n*_ = *U*_1−5_ in Fig. 5) is set at 1 eV. The graphene is furthermore assumed to be intrinsic, by which we mean that the Fermi level lies at the Dirac point at thermal equilibrium. The resulting curves capture well the conductance variations found in experiment (compare, for example, with the results of Fig. 3), showing a zero-bias anomaly that is suppressed with increasing voltage and temperature. The values of the characteristic voltage (~ mV) and temperature (> 40 K) required to suppress the anomaly are moreover consistent with the results of our experiment. To further highlight the extent of the agreement between experiment and theory, in the main panel of Fig. 6 we plot the result of rescaling the calculated conductance curves, using the same approach as that applied to the experimental data shown in Figs. 3, 4. Here, again, we find that the data conform to a universal voltage scaling, showing little change in differential conductance when *eV*/*k*_*B*_*T* ≲ 1, followed by a crossover to a logarithmically increasing conductance at larger voltages. Most importantly, the collapse of the temperature-dependent data onto a single curve in this figure, without the need to make use of any adjustable parameters, reproduces the key observation of our experiment. Overall, the suggestion is that our theoretical model therefore describes the essential physics exhibited in the experiment.

## Discussion

### Alternative Mechanisms

Thus far we have considered how an applied bias suppresses weak localization, purely in terms of the dispersion decoherence resulting from the presence of this bias. For completeness, however, one should also consider the possibility that the zero-bias anomaly results, instead, from the influence of electron heating, generated by this voltage. We have examined this possibility, however, and find that, while the applied bias does indeed give rise to some such heating, the magnitude of this effect is far too small to account for the observed conductance anomaly. This conclusion follows from studies^45^ in which we have utilized the mesoscopic fluctuations, observed in the low-temperature conductance of our devices (see Fig. 1(a)), as a hot-electron thermometer. These studies have shown that, with the lattice temperature maintained at a fixed value *T*_*L*_, the induced hot-electron temperature (*T*_*e*_) can be related to the dissipated electrical power (per electron, *P*_*e*_) as ${P}_{e}=\gamma ({T}_{e}^{3}-{T}_{L}^{3})$. For the samples that we have studied here, the prefactor *γ* is typically of order 2 × 10^3^ eV/sK^3^ ^45^. Noting that a bias of a few mV is sufficient to suppress the zero-bias anomaly in Figs. 2 & 3, and assuming a typical electron number of 10^4^ and a conductance of 10^−4^ S, we infer an induced hot-electron temperature of *T*_*e*_ =  7 K, relative to a fixed lattice temperature *T*_*L*_ =  3 K. The crucial point to note here is that, while the applied bias therefore does indeed induce some carrier heating, this effect is nonetheless relatively small, and far too weak to account for the observed conductance anomalies. This may be understood by focusing on the manner in which the quantum corrections are manifested in the zero-bias (linear) conductance, while varying the lattice (and thus, simultaneously, the electron) temperature. The temperature-dependent evolution of the zero-bias conductance in Fig. 3(a) indicates that *T*_*L*_ must be increased to beyond 30 K before the quantum corrections are fully suppressed, a conclusion that is supported by the results of Figs 1(a), which point to the survival of the corrections to even higher temperatures. Thus, it seems clear that the zero-bias anomaly cannot be primarily due to electron heating but must instead be dominated by an alternative mechanism. Based on the quantitative agreement between Figs. 3(b,c), 4(b,c) & 6, we suggest that this mechanism is that of dispersion decoherence described above.

The role of energy-averaging in mesoscopic transport has been highlighted previously, in discussions of the thermal damping of universal conductance fluctuations in dirty metals^67^. There, the averaging is described as an unavoidable source of static dephasing, arising from the thermal spread of the electron energy at nonzero temperature. In such systems, this static dephasing must be considered in addition to the dynamic dephasing^44^, generated by scattering from time-dependent sources (such as phonons and other electrons). One question that arises here is whether the observed features of the conductance anomaly really do result from energy averaging? An alternative scenario that might be considered is that application of the bias voltage instead increases dynamic dephasing, by enhancing electron-phonon and electron-electron scattering. This would likely lead to a power-law variation of the dynamic dephasing length (*l*_*φ*_ ∝ *V*^−*p*^, *p* ~ 1,^44^), and thus, once again, to a logarithmic conductance scaling. In such a situation, however, it is not at all clear that the resulting conductance variations should exhibit the universal voltage scaling found here. Specifically, the observation that the conductance curves collapse on one another when rescaled by the thermal energy, and that the rescaled data show a crossover in behaviour when *eV* ~ *k*_*B*_*T*, would appear to favor the energy-averaging picture presented here.

### Some Further Comments

In usual discussions of weak localization^3,67^, the static dephasing associated with thermal smearing is known to leave the localization unaffected. Physically, this result may be understood in the following way. At nonzero temperature, the backscattering responsible for weak localization can be attributed to a family of partial waves, with an energy spread set by the temperature. At each fixed energy within this range, a contribution to localization arises from the interference of time-reversed pairs of closed trajectories. Since each such pair returns to its origin in phase, subsequently summing over all relevant energies preserves the coherence required for the localization correction, and there is no thermal averaging of this effect. In our calculations of the energy averaging induced under nonequilibrium, however, the presence of a nonzero voltage *does* lead to averaging of the localization effect. Formally, this is due to the different way in which the various scattering diagrams are summed in the nonequilibrium model. Specifically, if we consider the contribution to localization generated by a specific set of impurities, at nonzero voltage this is calculated by first of all evaluating the (incoherent) interference between partial waves in the energy window set by the applied voltage, and which propagate in the same direction of time. Following this, a similar interference term is then calculated for the time-reversed counterparts of these waves. Finally, the localization contribution is determined by evaluating the interference between these two resultant waves. As the voltage is increased and the size of the energy window grows, this leads to self-averaging of the wavefunction as diffusing waves gradually decohere with one another as they propagate around the same loop (see Fig. 5).

There are two essential features of the conductance scaling found in our experiment that are reproduced by our nonequilibrium theory, giving confidence that it does indeed capture the essential behaviour underlying our observations. The first aspect is the identification of the dimensionless voltage (*eV*/*k*_*B*_*T*), which sets the bias scale for the onset of energy averaging. A similar term is often encountered when discussing the operation of various semiconductor devices, in which the role of the applied bias is to modulate the effective energy barrier within the device (think, for example, how an applied voltage modifies the depletion barrier that is present in either a *pn* junction or a bipolar transistor). In the case of interest here, however, the role of the voltage is somewhat different, opening a nonzero energy window within which different partial waves undergo quantum interference. The other feature of the scaling is the logarithmic variation of the conductance, observed in experiment once *eV*/*k*_*B*_*T* > 1. This behaviour, too, is reproduced by our theory, which identifies it as arising from the influence of dispersion decoherence on coherent backscattering.

While we have calculated the effect of energy averaging on the weak-localization correction alone, we know from our experiment (see Fig. 2) that the observed conductance anomaly arises from the combined influence of localization and electron interactions. While a theoretical treatment of the latter correction lies beyond the scope of the current work, the close similarity exhibited between our experimental and theoretical results suggests, at least, that the interactions should be subject to a similar energy averaging. Indeed, in a previous study of the differential conductance of metallic nanobridges, the observed zero-bias anomaly was attributed to a quenching of the interaction correction alone, rather than the influence of weak localization^36,37^. Our results for graphene clearly point to the combined influence of both mechanisms.

In previous explorations of the manner in which weak localization is affected under nonequilibrium conditions, a number of experiments have emphasized the inability of an electric field to break the time-reversal symmetry responsible for coherent backscattering^2,33,34,57^. We emphasize here that our findings do not contradict this principle; the suppression of the zero-bias anomaly that we observe is *not* a signature of breaking time reversal, but instead arises from the dispersion decoherence that we have described. A comment that should be made here concerns the apparent contradiction between our results and the original work of Bergmann^57^, who performed careful experiments in which the influence of field-induced heating could be discounted, and who showed that the localization correction was independent of electric field. (The maximum field applied in these studies was of order a few V/cm, which is comparable to the scale on which the quantum corrections are suppressed in our experiments.) We suggest that the primary reason that the influence of dispersion decoherence was not apparent in his experiments was likely that they were performed on large-area metal films, in which the inelastic scattering length would have been very much smaller than the sample size. Under such conditions, it is unlikely that the influence of the energy window defined by the applied voltage would have been experimentally resolved. In contrast, our experiments are performed on mesoscopic semiconductors in which the inelastic scattering length can be comparable to the sample dimensions^45^, allowing such effects to be observed.

Finally, we note that our calculations of the localization contribution are performed for intrinsic graphene, by which we mean that the Fermi level is taken to lie at the Dirac point at thermal equilibrium. As demonstrated in Figs. 3, 4, however, the bias-induced suppression of the quantum corrections, and the zero-bias anomaly that it leads to, appears to be a general feature of mesoscopic transport, for both electrons and holes in graphene. Similarly, while our model is formulated for a monolayer sheet of graphene, the observation of similar anomalies in our monolayer and bilayer devices point to the more general nature of the voltage-induced energy averaging. This conjecture is drawn from the fact that the weak localization phenomenon is not depending on the specific details of the energy dispersion relation but is rather a property related to the density of electron states in the two-dimensional material. It is, therefore, safe to presume that our calculations would yield a qualitatively analogous result also in the case of graphene bilayer. While in the absence of quantum corrections the temperature coefficient of the resistance is of opposite sign for the monolayer and bilayer, in the low-temperature regime where phase coherence is important both materials exhibit decreasing resistance with increasing temperature. In this limit, we find that this component of the resistance exhibits the universal scaling that we have observed here, for both monolayer and bilayer samples. To the extent that we have performed comparative studies of these samples, we have not been able to identify any quantitative difference in their quantum corrections.

## Conclusions

In conclusion, in this work we have explored the manner in which the quantum corrections to the low-temperature conductance of graphene, arising from weak localization and from electron interactions, are modified under nonequilibrium. In our studies of the differential conductance of monolayer and bilayer devices, we have demonstrated the presence of a zero-bias anomaly at low temperatures, which arises from a voltage-induced averaging of the quantum corrections. By implementing a simple rescaling of these data, in which we plot the bias-induced change of differential conductance as a function of the dimensionless voltage (*eV*/*k*_*B*_*T*), we have shown how this anomaly collapses onto a universal, temperature-independent form. According to this, the linear conductance remains approximately unchanged for voltages *eV* ≲ *k*_*B*_*T*, while at larger voltages it increases as a logarithmic function of *V*, reflecting the quenching of the quantum corrections. For insight into the origins of this behaviour, we have made use of nonequilibrium Green functions to formulate a formal description of weak-localization in the presence of nonequilibrium transport. According to this model, the voltage applied under nonequilibrium gives rise to an additional dephasing in transport, arising from a self-averaging effect. By establishing the manner in which the quantum corrections are suppressed in graphene, our study will be of broad relevance to the investigation of nonequilibrium transport in mesoscopic systems in general. This includes systems implemented from conventional metals and semiconductors, as well as those realized using other two-dimensional semiconductors^68,69^ and topological insulators^70,71^.

## Supplementary information

Supplementary Information

## Data Availability

All data generated and analysed during this study are included in this published article (and its Supplementary Information files).
