# Supplementary material for: Universal scaling of weak localization in graphene due to bias-induced dispersion decoherence
Source: Sci Rep. 2020 Mar 27;10:5611. doi: 10.1038/s41598-020-62313-3 (PMC7101405; doi:10.1038/s41598-020-62313-3)
Supplement: Supplementary file 1 — Supplementary Information [file 41598_2020_62313_MOESM1_ESM.pdf]

Supplementary Information: Universal scaling of weak localization in graphene due to bias-induced *dispersion decoherence*

R. Somphonsane*[1,2], H. Ramamoorthy[3], G. He[4], J. Nathawat[4], S. Yin[4], C.-P. Kwan[5], N. Arabchigavkani[5], B. Barut[5], M. Zhao[6], Z. Jin[6], J. Fransson[7], and J. P. Bird[4]

[1]Department of Physics, King Mongkut's Institute of Technology Ladkrabang, Bangkok 10520, Thailand
[2]Thailand Center of Excellence in Physics, Commission on Higher Education, 328 Si Ayutthaya Road, Bangkok 10400, Thailand
[3]Department of Electronic Engineering, King Mongkut's Institute of Technology Ladkrabang, Bangkok 10520, Thailand
[4]Department of Electrical Engineering, University at Buffalo, the State University of New York, Buffalo, NY 14260-1900, USA
[5]Department of Physics, University at Buffalo, the State University of New York, Buffalo, NY 14260-1500, USA
[6]High-Frequency High-Voltage Device and Integrated Circuits Center, Institute of Microelectronics of Chinese Academy of Sciences, 3 Beitucheng West Road, Chaoyang District, Beijing, PR China
[7]Department of Physics and Astronomy, Uppsala University, Box 516, SE-751 21 Uppsala, Sweden
*ratchanok.so@kmitl.ac.th

1 Breakdown of equilibrium theory

An approach for calculating the charge current, based on the real space distribution of the scattering impurities, can be formulated in terms of the current operator[2]

$$\mathbf{j}(\mathbf{r},t) \sim (-i) \lim_{\mathbf{r}'\to\mathbf{r}} \Big(\nabla_{\mathbf{r}} - \nabla_{\mathbf{r}'}\Big) \mathbf{G}^{<}(\mathbf{r},\mathbf{r}';t,t), \tag{1}$$

where $\mathbf{G}^{<}(\mathbf{r},\mathbf{r}';t,t)$ is the lesser form of the single-electron Green function for the electronic structure.

In a single-electron theory, assuming time-independent scattering potentials $\mathbf{V}_m \equiv \mathbf{V}(\mathbf{r}_m)$ at the spatial position $\mathbf{r}_m$, the contour-ordered Green function can be expanded according to ($x = (\mathbf{r},t)$)

$$\begin{aligned}
\mathbf{G}(x,x') =& \mathbf{G}_0(x,x') + \sum_m \int \mathbf{G}_0(x;\mathbf{r}_m,\tau)\mathbf{V}_m \mathbf{G}(\mathbf{r}_m,\tau;x')d\tau \\
=& \mathbf{G}_0(x,x') + \sum_m \int \mathbf{G}_0(x;\mathbf{r}_m,\tau)\mathbf{V}_m \mathbf{G}_0(\mathbf{r}_m,\tau;x')d\tau \\
&+ \sum_{mn} \int \mathbf{G}_0(x;\mathbf{r}_m,\tau)\mathbf{V}_m \mathbf{G}_0(\mathbf{r}_m,\tau;\mathbf{r}_n,\tau') \\
&\qquad \times \mathbf{V}_n \mathbf{G}_0(\mathbf{r}_n,\tau';x')d\tau d\tau' + \cdots ,
\end{aligned} \tag{2}$$

where $\mathbf{G}_0(x,x')$ is the bare Green function of the pristine system. While it is justified to assume spatial translational invariance of $\mathbf{G}_0$, in the presence of an electric field $\mathbf{E}$ there is a cubic time-dependence to the Green function, as can be seen by expressing it in its retarded form[3]

$$\mathbf{G}_0^r(\mathbf{k};t,t') = (-i)\theta(\tau)e^{-i(\varepsilon_{\mathbf{k}}\tau + \tilde{E}^2\tau^3)}, \tag{3}$$

where $\tau = t - t'$, $\varepsilon_{\mathbf{k}}$ is the single-electron energy at equilibrium, $\tilde{E}$ is an energy corresponding to the electric field $\mathbf{E}$, and d denotes the system dimensionality.

Upon conversion of the contour ordered expansion to the real time propagator $\mathbf{G}^<(x,x')$ we can make use of the Langreth rules[4]

$$\int A(t,\tau)B(\tau,t')d\tau \to \int \Big(A^r(t,\tau)B^<(\tau,t') + A^<(t,\tau)B^a(\tau,t')\Big)d\tau, \tag{4}$$

whenever $t < t'$ in contour sense. As we shall see below, the components of the expansion in Eq. (2) that contribute to weak localization have an odd number of propagators $\mathbf{G}_0$, for which the Langreth rules imply that (suppressing the spatial coordinates)

$$\begin{aligned}&\int \mathbf{G}_0(t,\tau)\mathbf{G}_0(\tau,\tau')\mathbf{G}_0(\tau',t')d\tau d\tau'\\&\to \int \Big(\mathbf{G}_0^r(t,\tau)\mathbf{G}_0^r(\tau,\tau')\mathbf{G}_0^<(\tau',t') + \mathbf{G}_0^r(t,\tau)\mathbf{G}_0^<(\tau,\tau')\mathbf{G}_0^a(\tau',t')\\&\quad + \mathbf{G}_0^<(t,\tau)\mathbf{G}_0^a(\tau,\tau')\mathbf{G}_0^a(\tau',t')\Big)d\tau d\tau',\ t<t'.\end{aligned} \tag{5}$$

As discussed in Ref.[3], the second term above provides the dominant contribution, allowing us to omit the first and last terms in the following discussion. In general, we can focus our efforts on contributions of the type $\mathbf{G}_0^r\cdots\mathbf{G}_0^r\mathbf{G}_0^<\mathbf{G}_0^a\cdots\mathbf{G}_0^a$, comprising an equal number of retarded Green functions to the left as advanced functions to the right. It should be noted that the linear-response result is derived from an analogous group of products, albeit without the presence of the lesser Green function. Hence, since the linear-response theory is local in time, the resulting time-integrals are trivial and this results in the cancelation of the electric field dependence noted previously[1].

In contrast to the equilibrium case, the presence of the lesser Green function in the nonlinear theory prevents any cancellation of the electric field dependence. Indeed, even when taking the simplest possible form of the lesser bare Green function, $\mathbf{G}_0^<(x,x') = (-i)\int f(\varepsilon_\mathbf{k})\exp\{-i\varepsilon_\mathbf{k}(t-t') + i\mathbf{k}\cdot(\mathbf{r}-\mathbf{r}')\}d\mathbf{k}/(2\pi)^d$, which corresponds to the case of a free electron gas in the absence of any external field, the three propagator product $\mathbf{G}_0^r\mathbf{G}_0^<\mathbf{G}_0^a$ results in the time-integration

$$\int_{-\infty}^{t}\int_{-\infty}^{t'} e^{-i\varepsilon_\mathbf{k}(t-\tau)-i\tilde{E}(t-\tau)^3-i\varepsilon_{\mathbf{k}'}(\tau-\tau')-i\varepsilon_{\mathbf{k}''}(\tau'-t')-i\tilde{E}(\tau'-t')^3}d\tau' d\tau. \tag{6}$$

Self-energy diagrams that describe the interference from which weak localization emerges comprise crossings of the impurity lines, see Fig. 1 (h), which convolutes the momentum variables such that the above time-integral would have to be written as

$$\begin{aligned}&\int_{-\infty}^{t}\int_{-\infty}^{t'} e^{-i\varepsilon_{\mathbf{p}/2+\kappa}(t-\tau)-i\varepsilon_\mathbf{q}(\tau-\tau')-i\varepsilon_{\mathbf{p}/2-\kappa}(\tau'-t')}\\&\qquad\times e^{-i\tilde{E}(t-\tau)^3-i\tilde{E}(\tau'-t')^3}d\tau' d\tau,\end{aligned} \tag{7}$$

where $\mathbf{p} = \mathbf{k}+\mathbf{q}$. Because of this convoluted structure of the self-energy in both momentum and time, it will be seen that the electric field cannot be removed from the interference factors.

Finally, we remark that the procedure described in this section is not viable for practical calculations. The cubic time dependence of the single-electron Green functions, for example, prevents a systematic order-by-order treatment of the physics involved. Consequently, we outline a different approach in the following sections that is a generalization of the concepts introduced in[5]. This approach allows us to discuss the propagators in the energy domain, under the assumption of stationary conditions, and thus simplifies the systematics of the calculations.

2 Model and transport formalism

We model the low-energy physics around the Fermi level of pristine graphene using the tight-binding Hamiltonian

$$\mathscr{H}_0 = -t\sum_{\langle ij\rangle\sigma} a_{i\sigma}^\dagger b_{j\sigma} + H.c., \tag{8}$$

where $a_{i\sigma}$ and $b_{j\sigma}$ denote the electron operators in the A- and B- sub-lattice, respectively. The nearest neighbor ($\langle ij\rangle$) intersite hopping rate is denoted by t. Assuming a spin-degenerate system, we can drop the spin subscript $\sigma = \uparrow,\downarrow$. We add a dilute random dispersion of impurities through

$$\mathscr{H}_I = \int \Psi^\dagger(\mathbf{r})\mathbf{V}(\mathbf{r})\Psi(\mathbf{r})d\mathbf{r}, \tag{9}$$

where $\Psi(\mathbf{r}) = \int \Psi_{\mathbf{k}} e^{-i\mathbf{k}\cdot\mathbf{r}} d\mathbf{k}/\rho$, $\Psi_{\mathbf{k}} = (a_{\mathbf{k}}\ b_{\mathbf{k}})^t$, ρ is the graphene planar density[8], and $\mathbf{V}(\mathbf{r}) = \sum_m \mathbf{V}_m \delta(\mathbf{r}-\mathbf{r}_m)$ denotes the scattering potential due to these impurities. Here, $\mathbf{V}_m = U\sum_m(\sigma_A \mathbb{1}_{m\in A} + \sigma_B \mathbb{1}_{m\in B})$ with $\sigma_A = (\sigma_0+\sigma_z)/2$ ($\sigma_B = (\sigma_0-\sigma_z)/2$), $\mathbb{1}_{m\in A(B)}$ is the indicator function for $\mathbf{r}_m$ within the A-sublattice (B-sublattice), and U is the scattering potential.

In reciprocal space, the model $\mathscr{H}_{\text{gr}} = \mathscr{H}_0 + \mathscr{H}_I$ is transformed into $\mathscr{H}_{\text{gr}} = \sum_{\mathbf{k}} \Psi^\dagger_{\mathbf{k}} \Phi_{\mathbf{k}} \Psi_{\mathbf{k}} + \sum_{\mathbf{k}\mathbf{k}'} \Psi^\dagger_{\mathbf{k}} \mathbf{V}_{\mathbf{k}\mathbf{k}'} \Psi_{\mathbf{k}'}$. Here

$$\Phi_{\mathbf{k}} = \begin{pmatrix} 0 & \phi_{\mathbf{k}} \\ \phi^*_{\mathbf{k}} & 0 \end{pmatrix}, \tag{10}$$

where the structure factor $\phi_{\mathbf{k}} = -t\sum_j \exp(i\mathbf{k}\cdot\delta_j)$ is given in terms of the nearest neighbor vectors $\delta_1 = a(\sqrt{3},1)/2$, $\delta_2 = -a(\sqrt{3},-1)/2$, and $\delta_3 = -a(0,1)$, with lattice parameter a. Free electrons have the dispersion relation $\phi_{\mathbf{k}\pm\mathbf{K}} \approx \pm v_F k e^{\pm i\varphi}$ around the K-points $\mathbf{K}_\pm = \pm\mathbf{K} = \pm 4\pi\sqrt{3}(1,0)/9a$, with Fermi velocity $v_F = 3at/2$. In $\mathbf{k}$-space, the scattering potential $\mathbf{V}_{\mathbf{k}\mathbf{k}'} = \sum_m \mathbf{V}_m \exp[-i(\mathbf{k}-\mathbf{k}')\cdot\mathbf{r}_m]/\Omega$, where Ω is the volume.

The conductance of the disordered graphene flake is calculated by placing it in the junction between a pair of metallic leads, modeled here with Hamiltonians $\mathscr{H}_L = \sum_{\mathbf{p}}(\varepsilon_{\mathbf{p}} - \mu_L) c^\dagger_{\mathbf{p}} c_{\mathbf{p}}$ and $\mathscr{H}_R = \sum_{\mathbf{q}}(\varepsilon_{\mathbf{p}} - \mu_R) c^\dagger_{\mathbf{q}} c_{\mathbf{q}}$, where the chemical potentials $\mu_{L/R}$ are related to the applied voltage V by $\mu_L - \mu_R = eV$. Tunneling between the leads and the graphene is described by the Hamiltonian $\mathscr{H}_T = \sum_{\mathbf{pk}} c^\dagger_{\mathbf{p}} t_{\mathbf{pk}} \Psi_{\mathbf{k}} + \sum_{\mathbf{qk}} c^\dagger_{\mathbf{q}} t_{\mathbf{qk}} \Psi_{\mathbf{k}} + H.c.$, where the row vector $t_{\mathbf{pk}}$ ($t_{\mathbf{qk}}$) denotes the tunneling rate between the left (right) lead and the graphene. It must be kept in mind that electrons in both sub-lattices take part in tunneling to and from the leads, which is accounted for here by the vectors $t_{\mathbf{pk}}$ and $t_{\mathbf{qk}}$.

The stationary charge current is given by $I = -e\partial_t \langle N_L\rangle = -e\partial_t \sum_{\mathbf{k}\sigma} \langle c^\dagger_{\mathbf{p}\sigma} c_{\mathbf{p}\sigma}\rangle$, which using standard methods becomes

$$I = \frac{ie}{h}\text{tr}\sum_{\mathbf{k}\mathbf{k}'}\int \Gamma^L_{\mathbf{k}\mathbf{k}'}\Big(f_L(\omega)\mathbf{G}^>_{\mathbf{k}'\mathbf{k}}(\omega) + f_L(-\omega)\mathbf{G}^<_{\mathbf{k}'\mathbf{k}}(\omega)\Big)d\omega. \tag{11}$$

Here, the trace runs over the pseudo-spin degrees of freedom, $f_\chi(x) = f(\omega-\mu_\chi)$ is the Fermi function at the chemical potential μ_χ, and $\Gamma^\chi_{\mathbf{k}\mathbf{k}'}$ is the coupling between the lead $\chi = L,R$ and the central region. We omit the momentum dependence of the coupling, $\Gamma^\chi_{\mathbf{k}\mathbf{k}'} = \Gamma^\chi$ and assume that the lesser/greater Green function for the central region $\mathbf{G}^{</>}_{\mathbf{k}\mathbf{k}'}(\omega) = \mathbf{G}^{</>}_{\mathbf{k}}(\omega)$.

3 Electronic structure calculation

We describe the weak-localization correction in graphene by considering the features of its electronic structure. In these calculations, the assumption of nonequilibrium conditions requires that we expand all calculated quantities on the Keldysh contour.

The electronic structure of pristine graphene is described by the *free* (unperturbed) graphene Green function $\mathbf{g}(\mathbf{k};z) = (z\sigma_0 + \Phi_{\mathbf{k}})(z^2 - |\phi_{\mathbf{k}}|^2)$, where $z \in \mathbb{C}$. We write the equation of motion for the Green function $\mathbf{G}(\mathbf{k},\mathbf{k}';z) = \langle\langle \Psi_{\mathbf{k}} | \Psi^\dagger_{\mathbf{k}'}\rangle\rangle(z)$ as the Dyson equation

$$\mathbf{G}_{\mathbf{k}\mathbf{k}'} = \delta_{\mathbf{k}\mathbf{k}'}\mathbf{g}_{\mathbf{k}} + \sum_\kappa \mathbf{g}_{\mathbf{k}}\mathbf{V}_{\mathbf{k}\kappa}\mathbf{G}_{\kappa\mathbf{k}'}, \tag{12}$$

and expand in orders of the scattering potential $\mathbf{V}_{\mathbf{k}\mathbf{k}'}$,

$$\mathbf{G}_{\mathbf{k}\mathbf{k}'} = \delta_{\mathbf{k}\mathbf{k}'}\mathbf{g}_{\mathbf{k}} + \mathbf{g}_{\mathbf{k}}\mathbf{V}_{\mathbf{k}\mathbf{k}'}\mathbf{g}_{\mathbf{k}'} + \sum_\kappa \mathbf{g}_{\mathbf{k}}\mathbf{V}_{\mathbf{k}\kappa}\mathbf{g}_\kappa\mathbf{V}_{\kappa\mathbf{k}'}\mathbf{g}_{\mathbf{k}'} + \cdots, \tag{13}$$

which enables an order-by-order investigation of the electronic structure in terms of the scattering potential.

3.1 Impurity averaging

We calculate the weak localization correction by making an average over impurities located at $\{\mathbf{r}_m\}$, thereby surrendering the non-locality of the Green function in reciprocal space. Employing the method outlined in[3,5], we obtain to first order

$$\overline{\mathbf{V}}_{\mathbf{k}\mathbf{k}'} = \frac{U}{\Omega}(N_A\sigma_A + N_B\sigma_B)\delta_{\mathbf{k}\mathbf{k}'}. \tag{14}$$

The Feynman diagram corresponding to this scattering process is depicted in Fig. 1 (a). Assuming equal numbers of impurities in the two sublattices, $N_A = N_B = N$, we can write

$$\overline{\mathbf{V}}_{\mathbf{k}\mathbf{k}'} = \frac{N}{\Omega}U\sigma_0\delta_{\mathbf{k}\mathbf{k}'} = cU\sigma_0\delta_{\mathbf{k}\mathbf{k}'}, \tag{15}$$

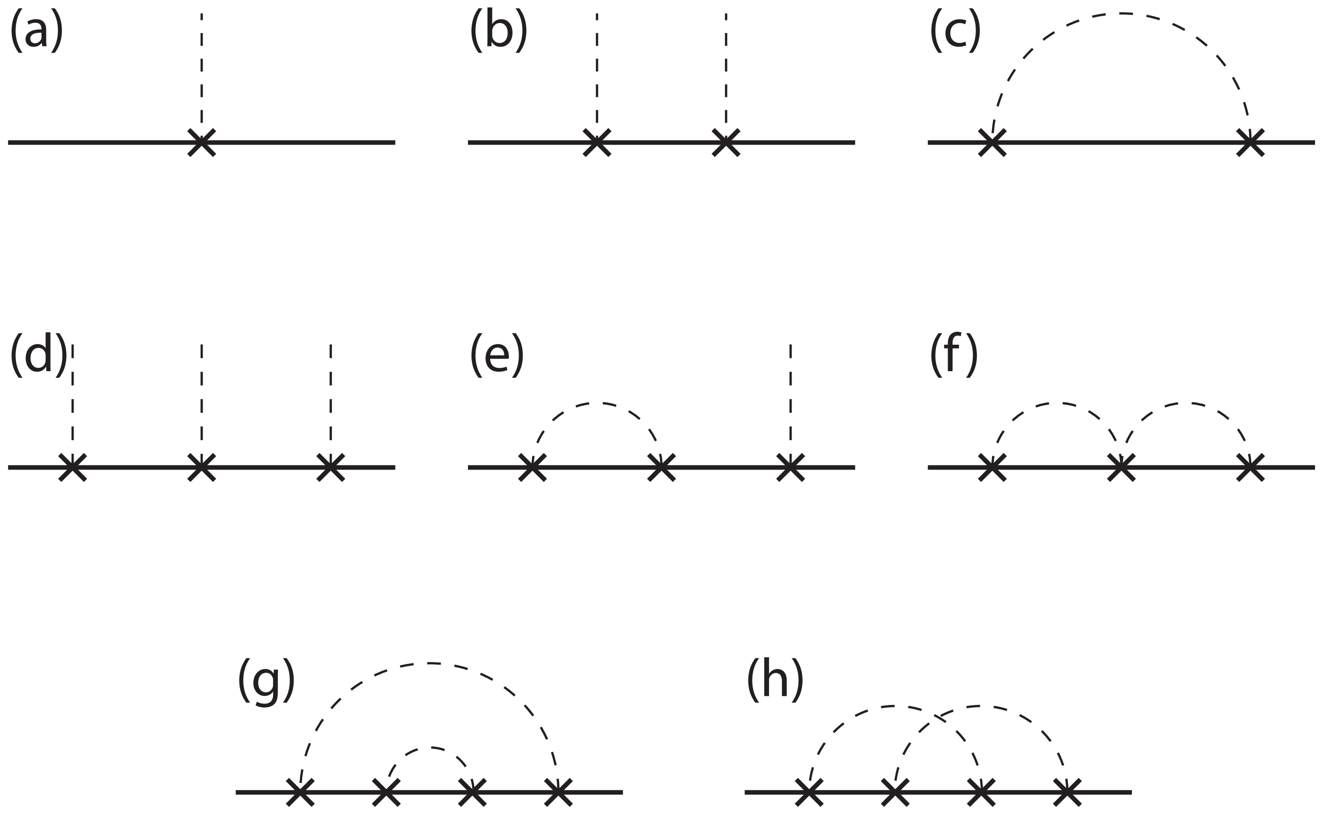

Figure 1. Some of the low-order diagrams that are important for conductivity calculations. Momentum is conserved at each vertex. Solid (dashed) lines represent free-electron Green function (impurity potential), whereas crosses mark scattering events. Diagrams (c), (f), and (g) are the lowest-order rainbow diagrams, whereas diagram (h) is the lowest-order crossed diagram.

where $c = N/\Omega$ defines the concentration of impurities. The first order correction to the Green function is, hence, given by

$$\delta \mathbf{G}^{(1)}_{\mathbf{k}} = cU\mathbf{g}^2_{\mathbf{k}}. \tag{16}$$

In an analogous manner, the second-order component becomes

$$\overline{\mathbf{V}_{\mathbf{k}\kappa}\mathbf{g}_\kappa\mathbf{V}_{\kappa\mathbf{k}'}} = \frac{NU^2}{\Omega^2}\left((N-1)\mathbf{g}_\kappa\sigma_0 + \sum_{i=A,B}\sigma_i\mathbf{g}_\kappa\sigma_i\right)\delta_{\mathbf{k}\mathbf{k}'}. \tag{17}$$

The Feynman diagrams corresponding to this second-order scattering process are depicted in Figs. 1 (b) and (c), respectively. The distribution of impurities between the two sub-lattices leads to a restricted contribution from $\sum_\kappa \mathbf{g}_\kappa$, picking out the diagonal components only. This structural organization is important for some of the higher-order contributions.

The second-order correction is summarized as

$$\delta \mathbf{G}^{(2)}_{\mathbf{k}} = c^2U^2\left[1-\frac{1}{N}\right]\mathbf{g}^3_{\mathbf{k}} + cU^2\mathbf{g}_{\mathbf{k}}\tilde{\mathbf{g}}\mathbf{g}_{\mathbf{k}}, \tag{18}$$

where $\tilde{\mathbf{g}} = \sum_{i=A,B}\sum_\kappa \sigma_i\mathbf{g}(\kappa)\sigma_i$. Here, the first term can be absorbed into the single-particle energy. The last term, however, provides the first diagram in the class of rainbow diagrams, see Fig. 1 (c). A partial summation over this class of diagrams leads to an electronic structure that is dependent upon the effective impurity-limited scattering lifetime τ_I. Therefore, we define the self-energy in the self-consistent Born approximation in terms of the impurity averaged Green function $\overline{\mathbf{G}}$ ($u^2 = cU^2$)

$$\Sigma = \frac{u^2}{\Omega}\sum_{i=A,B}\sum_{\mathbf{k}}\sigma_i\overline{\mathbf{G}}_\kappa\sigma_i, \tag{19}$$

where the impurity averaged Green function is given in terms of the Dyson equation $\overline{\mathbf{G}}_{\mathbf{k}} = (\mathbf{g}^{-1}(\mathbf{k}) - \Sigma[\overline{\mathbf{G}}])^{-1}$. The ansatz $\Sigma^r(\omega) = (\Lambda - i/2\tau_I)\sigma_0$ for the retarded self-energy, leads to

$$\frac{u^2}{\Omega}\sum_{i=A,B}\sum_{\mathbf{k}}\sigma_i\overline{\mathbf{G}}^r_{\mathbf{k}}\sigma_i$$
$$= -\frac{4u^2}{D_c^2}\left(\omega + \frac{i}{2\tau_I}\right)\left(\ln\frac{D_c}{|\omega + i/2\tau_I|} + i\frac{\pi}{2}\mathrm{sign}(\omega)\right)\sigma_0, \tag{20}$$

where $D_c^2 = 4\pi v_F^2\rho$ defines an upper energy cut. Equating for Λ and $1/\tau_I$, and assuming that $1/\tau_I \ll |\omega|$ (which is sufficient for small energies around the Fermi level, since $D_c \sim 1$ eV, and $U \sim 1eV$, while $u^2/D_c^2 \sim 10^{-3}$ — 10^{-2}, implying that $1 - 4(u^2/D_c^2)\ln(D_c/|\omega + i/2\tau_I|) \geq 1$), leads to the result that we can neglect Λ, and retain only the approximate inverse lifetime, or, momentum scattering time

$$\frac{1}{\tau_I} \approx \frac{4\pi u^2}{D_c^2}|\omega|, \tag{21}$$

in agreement with previous studies[6,7].

3.2 Maximally-crossed diagrams

The weak localization phenomenon arises from enhanced backscattering, caused by the constructive interference between pairs of time-reversed closed paths. It is well known that diagrams containing crossed impurity lines account for this interference and that the largest contribution from this class is provided by the subclass of maximally-crossed diagrams. The diagram in Fig. 1 (h), which represents the lowest-order maximally-crossed diagram and can be written algebraically as

$$\Sigma^{(1)}_{cr}(\mathbf{k}) = \frac{1}{\Omega^4}\sum_{m\neq n}\sum_{\mathbf{q}\kappa}\mathbf{V}_m\overline{\mathbf{G}}_{\mathbf{p}/2+\kappa}\mathbf{V}_n\overline{\mathbf{G}}_{\mathbf{q}}\mathbf{V}_m\overline{\mathbf{G}}_{\mathbf{p}/2-\kappa}\mathbf{V}_n$$
$$\approx \frac{u^4}{\Omega^2}\sum_{\mathbf{q}\kappa}\sum_{i,j=A,B}\sigma_i\overline{\mathbf{G}}_{\mathbf{p}/2+\kappa}\sigma_j\overline{\mathbf{G}}_{\mathbf{q}}\sigma_i\overline{\mathbf{G}}_{\mathbf{p}/2-\kappa}\sigma_j, \tag{22}$$

where $\mathbf{p} = \mathbf{k} + \mathbf{q}$. The dominant contribution to the lesser/greater form of the self-energy is then given by[3]

$$\Sigma^{(1)</>}_{cr}(\mathbf{k}) = \frac{u^4}{\Omega^2}\sum_{\mathbf{q}\kappa}\sum_{i,j=A,B}\sigma_i\overline{\mathbf{G}}^r_{\mathbf{p}/2+\kappa}\sigma_j\overline{\mathbf{G}}^{</>}_{\mathbf{q}}\sigma_i\overline{\mathbf{G}}^a_{\mathbf{p}/2-\kappa}\sigma_j. \tag{23}$$

The matrices σ_i, $i = A,B$, are projections and orthogonal ($\sigma_i\sigma_j = \delta_{ij}\sigma_i$). For a general 2×2 matrix $\mathbf{A}$, we have $\sigma_A\mathbf{A}\sigma_A = A_{11}\sigma_A$ and $\sigma_B\mathbf{A}\sigma_B = A_{22}\sigma_B$, while $\sigma_A\mathbf{A}\sigma_B = A_{12}\sigma_+$ and $\sigma_B\mathbf{A}\sigma_A = A_{21}\sigma_-$, where $\sigma_\pm = (\sigma_x \pm i\sigma_y)/2$. Hence, a product on the form $\sigma_i\mathbf{A}\sigma_j\mathbf{B}\sigma_i\mathbf{C}\sigma_j$ reduces to

$$\sum_{i,j=A,B}\sigma_i\mathbf{A}\sigma_j\mathbf{B}\sigma_i\mathbf{C}\sigma_j = \begin{pmatrix} A_{11}B_{11}C_{11} & A_{12}B_{21}C_{12} \\ A_{21}B_{12}C_{21} & A_{22}B_{22}C_{22}\end{pmatrix}. \tag{24}$$

This property enables us to evaluate all of the crossed diagrams, element by element.

Since the diagonal components of the graphene Green function are equal, we only have to consider one of them. An analogous observation holds for the off-diagonal components. The calculation of all matrix components in the self-energy is fundamentally important since the coupling between the pseudo-spin degrees of freedom plays a central role in the theory of weak localization in graphene[?]. Hence, we write

$$\left(\Sigma^{(1)</>}_{cr}(\mathbf{k})\right)_{ij} = \frac{u^2}{\Omega}\sum_{\mathbf{q}}\zeta_{ij}(\mathbf{p})\left(\overline{\mathbf{G}}^{</>}_{\mathbf{q}}\right)_{ji}, \tag{25}$$

where the subscripts ij refer to matrix components and where

$$\zeta_{ij}(\mathbf{p}) = \frac{u^2}{\Omega}\sum_{\kappa}\left(\overline{\mathbf{G}}^r_{\mathbf{p}/2+\kappa}\right)_{ij}\left(\overline{\mathbf{G}}^a_{\mathbf{p}/2-\kappa}\right)_{ij}. \tag{26}$$

The momentum summation contained in ζ_{ij} is carried out by individually expanding the Green functions around the nodes in the two valleys $\pm\mathbf{K}$, such that both intra- and inter-valley scattering is included. Hence,

$$\phi_{\mathbf{p}/2\pm\kappa+s\mathbf{K}} \approx s\frac{v_F}{2}(pe^{is\varphi_{\mathbf{p}}} \pm 2\kappa e^{is\varphi_\kappa}),\ s=\pm, \tag{27}$$

where $\tan\varphi_{\mathbf{k}} = k_y/k_x$ and $\tan\varphi_{\mathbf{p}} = p_y/p_x$. This leads to the result that

$$\phi_{\mathbf{p}/2+\kappa}\phi_{\mathbf{p}/2-\kappa} \approx -E^2\sin^2\varphi_{\mathbf{p}} + 4\varepsilon^2\sin^2\varphi_\kappa, \tag{28}$$

where $E = v_F p$ and $\varepsilon = v_F\kappa$. Moreover, since $|\phi_{\mathbf{p}/2\pm\kappa}| \to v_F|\mathbf{p}/2\pm\kappa|$ in the valleys, and restricting ourselves to the regime $E \ll \varepsilon_F$, we can employ the approximation $v_F^2|\mathbf{p}/2\pm\kappa|^2 \approx \varepsilon^2 \pm \varepsilon_F E\cos\gamma$. In the notation $(z_\mp^{r/a})^2 = (z^{r/a})^2 \mp \varepsilon_F E\cos\gamma$, with $z^{r/a} = \omega \pm i/2\tau$ and $\gamma = \varphi_\kappa - \varphi_{\mathbf{p}}$, we have

$$\zeta_{11}(\mathbf{p}) = \frac{8u^2}{D_c^2}\int \frac{|z^r|^2}{[(z_-^r)^2-\varepsilon^2][(z_+^a)^2-\varepsilon^2]}\frac{\varepsilon d\varepsilon d\varphi_\kappa}{2\pi}, \tag{29a}$$

$$\zeta_{12}(\mathbf{p}) = -\frac{2u^2}{D_c^2}\int \frac{E^2\sin^2\varphi_{\mathbf{p}} - 4\varepsilon^2\sin^2\varphi_\kappa}{[(z_-^r)^2-\varepsilon^2][(z_+^a)^2-\varepsilon^2]}\frac{\varepsilon d\varepsilon d\varphi_\kappa}{2\pi}. \tag{29b}$$

The energy (ε) integration generates the contribution $\log[D_c^2/(iz_-^r)^2] - \log[D_c^2/(iz_+^a)^2] \approx i2\pi$ in both integrals, while ζ_{12} also contains the contribution

$$\log\frac{D_c^4}{(z_-^r)^2(z_+^a)^2} \approx 4\ln\frac{D_c}{|\omega|}. \tag{30}$$

In these equations, we have omitted any angular dependence in the logarithms, which in the latter case implies that $(z_-^r)^2(z_+^a)^2 \propto \omega^4$.

We can now write the kernels ζ_{ij} according to

$$\zeta_{11}(\mathbf{p}) = i\frac{2\pi u^2}{D_c^2}\int \frac{|z^r|^2}{i\omega/\tau - \varepsilon_F E\cos\gamma}\frac{d\varphi_\kappa}{2\pi}, \tag{31a}$$

$$\zeta_{12}(\mathbf{p}) = -\frac{u^2}{D_c^2}\int\left(4\sin^2\varphi_\kappa\ln\frac{D_c}{|\omega|} - i\pi\frac{E^2\sin^2\varphi_{\mathbf{p}} - 4\omega^2\sin^2\varphi_\kappa}{i\omega/\tau - \varepsilon_F E\cos\gamma}\right)\frac{d\varphi_\kappa}{2\pi}. \tag{31b}$$

Then, by performing the angular integration, we arrive at

$$\zeta_{11}(\mathbf{p}) = \frac{2\pi u^2}{D_c^2}\frac{\omega^2}{\sqrt{(\omega/\tau)^2 + (\varepsilon_F E)^2}}, \tag{32a}$$

$$\zeta_{12}(\mathbf{p}) = -\frac{u^2}{D_c^2}\left(2\ln\frac{D_c}{|\omega|} - \pi\frac{E^2 - 4\omega^2}{\sqrt{(\omega/\tau)^2 + (\varepsilon_F E)^2}}\sin^2\varphi_{\mathbf{p}}\right), \tag{32b}$$

where in the result for ζ_{11} we have replaced $|z^r|$ by ω (since $1/4\tau^2 \ll \omega^2$) and in that for ζ_{12} we have neglected a minor contribution proportional to ω^2. In fact, the second contribution to ζ_{12} can also be omitted without changing its vital properties pertaining to weak localization.

To first order in the self-energy, our calculation of the maximally-crossed diagrams yields the expression

$$\Sigma_{cr}^{(1)</>}(\mathbf{k}) = \frac{u^2}{\Omega}\sum_{\mathbf{q}}\left\{\frac{2\pi u^2\omega^2/D_c^2}{\sqrt{(\omega/\tau)^2+(\varepsilon_F E)^2}}\begin{pmatrix}\left(\overline{\mathbf{G}}_{\mathbf{q}}^{</>}\right)_{11} & 0 \\ 0 & \left(\overline{\mathbf{G}}_{\mathbf{q}}^{</>}\right)_{22}\end{pmatrix} - \frac{2u^2}{D_c^2}\ln\frac{D_c}{|\omega|}\begin{pmatrix}0 & \left(\overline{\mathbf{G}}_{\mathbf{q}}^{</>}\right)_{21} \\ \left(\overline{\mathbf{G}}_{\mathbf{q}}^{</>}\right)_{12} & 0\end{pmatrix}\right\}. \tag{33}$$

The result in Eq. (24) implies that the nth contribution to both diagonal and off-diagonal entries equal the nth power of the entries in the expression above. Hence, the summation over the maximally-crossed diagrams can be performed independently, in each matrix entry, giving

$$\sum_{n=1}^{\infty}\Big(\zeta_{11}(\mathbf{p})\Big)^n=\sum_{n=1}^{\infty}\Big(\zeta_{22}(\mathbf{p})\Big)^n\approx\frac{(\omega/\tau\varepsilon_F)^2}{E^2+(\omega/\tau\varepsilon_F)^2}, \tag{34a}$$

$$\sum_{n=1}^{\infty}\Big(\zeta_{12}(\mathbf{p})\Big)^n=\sum_{n=1}^{\infty}\Big(\zeta_{21}(\mathbf{p})\Big)^n\approx-\frac{2u^2}{D_c^2}\ln\frac{D_c}{|\omega|}. \tag{34b}$$

In the expressions for ζ_{11} and ζ_{22}, we have made use of the relation $4\pi u^2/D_c^2\approx 1/\tau|\omega|$. The self-energy contribution from the maximally-crossed diagrams is then reduced to the expression

$$\Sigma_{cr}^{</>}(\mathbf{k})=\frac{u^2}{\Omega}\sum_{\mathbf{q}}\left\{\frac{(\omega/\tau\varepsilon_F)^2}{E^2+(\omega/\tau\varepsilon_F)^2}\begin{pmatrix}\Big(\overline{\mathbf{G}}_{\mathbf{q}}^{</>}\Big)_{11} & 0\\ 0 & \Big(\overline{\mathbf{G}}_{\mathbf{q}}^{</>}\Big)_{22}\end{pmatrix}\right.$$
$$\left.-\frac{2u^2}{D_c^2}\ln\frac{D_c}{|\omega|}\begin{pmatrix}0 & \Big(\overline{\mathbf{G}}_{\mathbf{q}}^{</>}\Big)_{21}\\ \Big(\overline{\mathbf{G}}_{\mathbf{q}}^{</>}\Big)_{12} & 0\end{pmatrix}\right\}. \tag{35}$$

Next, we sum over the momentum $\mathbf{q}$. In the diagonal components, we note that $1/[E^2+(\tau\varepsilon_F/\omega)^2]$ is strongly peaked around $\mathbf{q}=-\mathbf{k}$, and recall that $E=v_F|\mathbf{k}+\mathbf{q}|$, which allows us to move $(\overline{\mathbf{G}}_{\mathbf{q}}^{</>})_{11(22)}$ out of the summation, the remainder of which yields a factor $\approx u^2$. The summation of the off-diagonal components is trivial since the contribution from ζ_{12} is independent of $\mathbf{q}$. Setting $\overline{\mathbf{G}}_{\mathbf{q}}^{</>}=\overline{\mathbf{G}}_{\mathbf{q}}^{r}\Sigma_0^{</>}\overline{\mathbf{G}}_{\mathbf{q}}^{a}$, where $\Sigma_0^{</>}(\omega)=(\pm i)\sum_\chi\Gamma^\chi f_\chi(\pm\omega)(\sigma_0+\sigma_x)$ leads to

$$\Sigma_{cr}^{</>}(\mathbf{k})\approx(\pm i)\sum_{\chi}\Gamma^\chi f_\chi(\pm\omega)\left[\sigma_0 u^2\left|\Big(\overline{\mathbf{G}}_{-\mathbf{k}}^{r}\Big)_{11}+\Big(\overline{\mathbf{G}}_{-\mathbf{k}}^{r}\Big)_{12}\right|^2\right.$$
$$\left.-\sigma_x\frac{3u^2}{D_c^2}\ln\frac{D_c}{|\omega|}\right]. \tag{36}$$

The diagonal components in this expression provide a contribution to the current that is quartic in the Green functions, which should be compared with the quadratic contribution generated by the off-diagonal terms. Since the multiplying factor is also constant, when compared to the logarithmic functions in the off-diagonal components, the diagonal components can be discarded when calculating the weak-localization correction. The off diagonal components account for the coupling between electrons in different sublattices, indicating that the pseudo-spin chirality is of great importance for the emergence of weak localization in graphene.

Finally, calculation of the differential conductance reduces to the procedure of taking the derivative of the relevant Fermi functions, yielding the correction

$$\frac{d\delta I}{dV}=-\frac{2e^2}{h}\Gamma^L\Gamma^R\int\mathscr{F}_V(\omega)\frac{1}{D_c^2}\ln\frac{D_c}{|\omega|}d\omega. \tag{37}$$

At low temperatures, this displays the logarithmic variation normally associated with weak localization in linear transport, since $\mathscr{F}_V(\omega)$ $\{=(\beta/4)\cosh^{-2}\beta(\omega-eV)/2\}\to\delta(\omega-eV)$, as $T\to 0$, giving

$$\frac{d\delta I}{dV}\to-\frac{2e^2}{h}\cdot\frac{\Gamma^L\Gamma^R}{D_c^2}\ln\frac{D_c}{|eV|},\ T\to 0. \tag{38}$$

References

1. G. Bergmann, Z. Phys. B **49**, 133 (1982).
2. L. P. Kadanoff and G. Baym, Quantum Statistical Mechanics (W. A. Benjamin, Inc., New York, 1962).
3. H. Haug and A. -P. Jauho, in Quantum Kinetics in Transport and Optics of Semiconductors (Springer-Verlag, Berlin/Heidelberg/New York, 1998).

4. D. C. Langreth, Linear and Nonlinear Electron Transport in Solids, ed. by J. T. Devreese and E. van Doren (Plenum, New York, 1976).

5. N. H. Shon and A. Ferraz, Sol. State Comm. **86**, 195 (1993).

6. N. H. Shon and T. Ando, J. Phys. Soc. Jpn. **67**, 2421 (1998).

7. H. Suzuura and T. Ando, Phys. Rev. Lett. **89**, 266603 (2002).

8. N. M. R. Peres, F. Guinea, and A. H. Castro Neto, Phys. Rev. B **73**, 125411 (2006).
